# A Bilobed, or Sacculated Bladder

**Published:** 1852-01

**Authors:** Thomas J. McKie

**Affiliations:** Woodlawn, S. C.


*A Bilobed, or Sacculated Bladder.* By Thomas J. McKie, M. D., of Woodlawn, S. C.—March 25th, 1851.—Isaac, a negro man, æt. 60 or 65 years, a common field hand, applied to me, complaining that he could not void his urine except in very small quantities at long intervals. Upon examination I found that he had had occasional attacks of this kind for six or eight months past, lasting variously from three days to a week; though without impairing materially his general health. He stated that he had always relieved himself, until now, by drinking freely of water-melon seed tea, without troubling his owner.

Previous to the origin of these attacks, he had been a remarkably healthy negro, notwithstanding he had exposed himself to every vicissitude of weather; having in his younger days, been a great runaway.

In his right groin he bore one or two large cicatrices which he said was the result of an injury received when a small boy; farther particulars of these scars he did not give, nor could he call to mind any other injury received since, to which his present distress might be attributed.

He was not subjected to any treatment previous to my seeing him, not making much complaint, and using his favorite tea (as stated) with never-failing relief. Acting upon this suggestion, I gave him more active diuretics; but with marked increase of suffering. Anti-spasmodics were then tried with no other effect than a partial relief of pain. Emetics were not more successful. By this time, the hypogastrium, and even the region of the umbilicus were made prominent by the greatly distended bladder.

An attempt was now made to introduce the catheter, but after several trials it was entirely unsuccessful. The silver, as well as different sizes of gum-elastic catheters were used.

His pulse soon becoming very full, and bounding, he was freely bled from the arm, which gave great relief, with passage of small quantities of urine. Patient seemed to be improving slowly for three days; pulse was more natural, and he voided a few drops of urine once or twice in twenty-four hours. About the end of the third day, patient's condition seemed to be getting worse again, when an examination was made by the rectum, and the prostate gland was found so enlarged as almost to prevent the passage of fecal matter, except in a very fluid state. Under the circumstances, thought myself justifiable in attempting to force a passage through the substance of the gland with the common silver catheter; but as in the previous attempt at introduction, I was uusuccessful.—The bladder still continued distended to its farthest limits, being partially relieved once or twice in twenty-four hours by the escape of a few drops of apparently healthy urine. Gave ext. hemp, acet. morphia, and ext. hyosci. alternately to alleviate pain, which was extreme, particularly in the afternoon and night, he always suffering much less in the morning.

March 29th. Patient began to decline very rapidly, vomiting almost incessantly, (and without any seeming effort) a matter very like urine in appearance, only of more consistency ; pulse remained rather frequent, with nearly natural skin, and a perfectly clear head.

At 3 o'clock, P. M., I very reluctantly punctured the bladder above the pubis, with a common flat trocar, without previous incision. A considerable quantity of healthy urine escaped, which gave the patient great relief, so that he rested well through the night.

30th. Patient apparently better; has no pain, though he sighs very often, and is troubled with hiccough ; is not restless, has some appetite ; water still dripples through the opening in the abdomen ; bowels moved naturally : at night gave one and a half grains ext. hemp, with five of ext. hyosciamus.

31st. No perceptible improvement since yesterday ; passed healthy urine per vias naturales ; opening in abdomen allowed to close. Gave eight grains ext. hyosci.

April 1st. No change, only makes a greater quantity of urine than usual. 2d. Patient the same. 3d. Grows more feeble, allowed him bread and wine.

April 4. Patient is still weaker, but does not complain of any particular pain, nor has he since being tapped, though he still continues to sigh very often.

P. M. Isaac is much worse, is evidently sinking fast. Before this change, he passed a large quantity of urine, which I mistook for a favorable indication. Death seems inevitable. April 5th. At 5, A. M., patient died, after a sleepless night ; he remained perfectly conscious of all around, and of his approaching end, till a few minutes before his death. At 2, P. M., made a post-mortem inspection. Introduced (without the slightest difficulty) the catheter, and drew off a large quantity of healthy urine. Made an incision along the linea alba, and another across it reaching from the right to the left lumbar rigion. The bladder was situated high up in the pelvis, and very strongly bound by extensive unnatural adhesions, to the parts adjoining. On dividing the wall of the bladder, it was found much thickened, and the inner coat covered with spots of effused blood, varying in size from a mustard seed to a large pea. The bladder itself was composed of *two distinct cavities* connected by a round opening about the size of the point of a finger. The larger one, or bladder proper, was situated in front and rather above the other, and was the one into which the ureters entered, as well as from which the urethra arose. The smaller one was situated behind and rather to the left, and was about one third the size of the other, of much thinner walls, and having but few muscular fibres, which seemed to be sent off from the larger one, consequently possessing but little power of contracting itself; a power which the other, or a larger, seemed to possess in an eminent degree.

The ureters were much dilated; the kidneys were also found extensively diseased, being much softened and entirely covered by a substance nearly resembling coagulated blood, but of firmer consistence, and about half an inch in thickness, extending on both sides far down the psoas muscles, to which it was attached, as well as to the small intestine and to the side of the abdomen.

Disease of kidneys was never suspected; the whole trouble having been charged entirely to the prostate gland, which I think was the principal cause of this anomalous case.

This singular bladder must have been the result of injury received by the patient when young, which ruptured its external coat; the dilatation of the internal, I suppose has increased with his years, particularly during the six months previous to his death.—*Charleston Med. Journal and Review.*

